# Focal Pancreatic Parenchymal Atrophy in Main Duct Intraductal Papillary Mucinous Neoplasms of the Pancreas

**DOI:** 10.7759/cureus.98506

**Published:** 2025-12-05

**Authors:** Hidehito Sumiya, Shinsuke Koshita, Yoshihide Kanno, Takahisa Ogawa, Hiroaki Kusunose, Toshitaka Sakai, Masaya Oikawa, Takashi Sawai, Yutaka Noda, Kei Ito

**Affiliations:** 1 Gastroenterology, Sendai City Medical Center, Sendai, JPN; 2 Surgery, Sendai City Medical Center, Sendai, JPN; 3 Pathology, Sendai City Medical Center, Sendai, JPN

**Keywords:** computed tomography, focal pancreatic parenchymal atrophy, intraductal papillary mucinous neoplasms, main duct type, pancreatic cancer

## Abstract

Introduction

Although upstream pancreatic atrophy (UPA) is a characteristic finding in main duct intraductal papillary mucinous neoplasms (MD-IPMNs) of the pancreas, the relationship between MD-IPMNs and focal pancreatic parenchymal atrophy (FPPA), which is an indicator of early-stage pancreatic ductal adenocarcinoma, remains unclear. Thus, this study aimed to investigate the relationship.

Methods

From the 49 patients diagnosed with MD-IPMNs using the resected specimens from June 2003 to December 2023, 19 patients were selected to clarify the clinical characteristics of FPPA for MD-IPMN patients. The primary outcome measure was the frequency of FPPA/UPA. Secondary outcome measures were (1) the locational relationship between MD-IPMN and FPPA and (2) the clinicopathological differences between those atrophic types.

Results

FPPA and UPA were observed in 4 (21%) and 12 (63%) patients, respectively. When the clinicopathological factors of FPPA and UPA patients were compared, only the diameter of the main pancreatic duct (MPD) significantly differed (median MPD diameter: 6 mm vs. 11 mm, p = 0.017). Of the four patients with FPPA, the histological extent of the MD-IPMN lesion was within that of FPPA in three patients (75%) and was concordant with that of FPPA only in one patient (25%).

Conclusions

FPPAs were detected in approximately 20% of resected MD-IPMNs, and 75% of those MD-IPMNs were histologically included in the area of the FPPAs. Since the MPD diameter was significantly smaller for MD-IPMNs with FPPAs than those with UPAs, MD-IPMNs with FPPAs may be biologically related to the mild dilation of the MPD, such as lesions at an early stage or less mucin secretion.

## Introduction

Intraductal papillary mucinous neoplasms of the pancreas (IPMNs) are mucin-producing intraductal epithelial neoplasms with dilated main and/or branch pancreatic ducts. IPMNs are classified into the following three types: branch duct IPMN (BD-IPMN), main duct IPMN (MD-IPMN), and mixed IPMN. BD-IPMN is defined as a pancreatic cyst >5 mm in diameter that communicates with the main pancreatic duct (MPD). MD-IPMN is characterized by segmental or diffuse dilation of the MPD >5 mm without other causes of obstruction. Mixed IPMN can be classified when it meets both the criteria of BD-IPMN and MD-IPMN [1,2]. According to the international consensus guidelines, the mean malignancy rate for resected MD-IPMNs is high, ranging from 36% to 100% [1,2]. Therefore, patients with MD-IPMNs are likely to undergo surgery in clinical practice due to the high risk of malignancy. On the other hand, some issues related to the diagnosis of MD-IPMNs remain unsolved as follows: (1) little have been known about characteristics of MD-IPMNs at an initial stage which can contribute to the early diagnosis of those; (2) a significant portion of resected MD-IPMNs are benign-about 40% in one study, even with mural nodules (MNs) [3]; and (3) evaluating the extension of MD-IPMN lesions along the MPD is not so easy, even using a recent digital scope for pancreatoscopy [4].

Upstream pancreatic atrophy (UPA) is a characteristic imaging finding of MD-IPMNs. Ban et al. have proposed an etiological mechanism for UPA as follows: excessive mucin secretion from IPMNs into the MPD prevents the flow of pancreatic juice, leading to upstream fibrosis and/or atrophy of the pancreatic parenchyma at the side of the pancreatic tail [5]. In addition, some researchers have reported that UPA is a predictive factor for malignant MD-IPMNs [6-8]. On the other hand, focal pancreatic parenchymal atrophy (FPPA) has recently been reported as a possible imaging finding for diagnosing pancreatic ductal adenocarcinoma (PDAC) at an early stage or pancreatic intraductal neoplasm (PanIN) [9-11]. In addition, the extension of this finding appears to be somewhat coincident with the lateral spreading of cancer epithelium [9-11].

Recently, Kikuyama et al. have reported the relationship between pancreatic cystic lesions, including IPMN and FPPA, and the results of pancreatic juice aspiration cytologic examination [12]. On the other hand, there have been few reports on the association between FPPA and MD-IPMNs. Considering the similarity in the intraductal neoplasms between MD-IPMN and PanIN, we hypothesized that there might be some kind of clinical or histological relationship between FPPA and MD-IPMN. In this study, we aimed to clarify the clinical significance of FPPA among MD-IPMN patients, especially focusing on the possible clinical contribution of FPPA to the detection of MD-IPMNs.

## Materials and methods

Study design and ethics statement

This retrospective study was conducted at Sendai City Medical Center and approved by its institutional review board (approval number: 2024-0014). Written informed consent was obtained from all patients related to this study.

Patients

**Figure 1:**
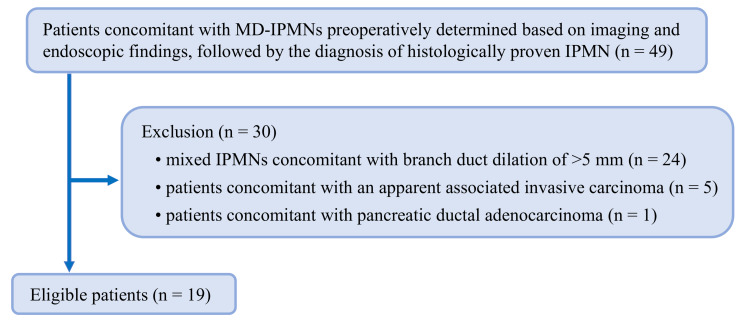
Flowchart outlining the study protocol. MD-IPMNs: main duct intraductal papillary mucinous neoplasms of the pancreas.

From the pathological prospectively registered database in our hospital, forty-nine consecutive patients who were histologically diagnosed with MD-IPMNs by analyzing the resected pancreatic specimens (i.e., IPMNs in which the neoplastic epithelium was mainly located in the MPD) between June 2003 and December 2023 were obtained. To recognize FPPA accurately using the imaging studies and resected pancreatic specimens, we determined invasive cancer in which invasive components could be detected using imaging studies as one of the exclusion criteria for the following reasons: (1) cancer invasion to the MPD often causes the pancreatic parenchymal atrophy on the upstream side of the MPD, and (2) invasive components can mask the atrophy of the pancreatic parenchyma. In addition, since the dilated branch ducts may prevent the accurate analysis on the pancreatic parenchymal atrophy, MD-IPMN with the dilated branch ducts of ≥5 mm were excluded. Accordingly, the exclusion criteria were determined as follows: (1) Mixed IPMNs with dilated branch ducts of >5 mm determined by using imaging studies, (2) IPMN-derived invasive carcinomas in which mass lesions could be detected preoperatively, and (3) IPMN-concomitant PDACs. Finally, “pure” MD-IPMNs, which met the criteria of MD-IPMN according to international guidelines, accounted for the majority of subjects in this study, and 19 patients were selected for the analyses (Figure 1).

Outcome measures

The primary outcome measure was the frequency of FPPA/UPA among MD-IPMN patients. Secondary outcome measures were (1) the locational relationship between MD-IPMNs and FPPAs, and (2) the clinicopathological differences between MD-IPMN patients with FPPA and those with UPA. Since MD-IPMNs are often associated with UPA, which has a higher incidence of malignancy [6-8], FPPA and UPA patients were compared to clarify the distinct features of FPPA.

For analyses of outcome measurements, the locational relationship between MD-IPMNs and FPPAs was histologically assessed using resected specimens. In addition, we retrospectively examined demographic, clinical, and imaging candidate factors. For demographic and clinical factors, age, sex, symptoms, such as abdominal pain and icterus, the presence of diabetes mellitus, history of acute pancreatitis, laboratory data including serum amylase, lipase, carbohydrate antigen 19-9 (CA19-9), and carcinoembryonic antigen levels were examined. Imaging factors, such as the location of MD-IPMNs, the diameter of the MPD, and the height of MNs, were evaluated by using contrast-enhanced computed tomography (CECT) or endoscopic ultrasonography (EUS). The MPD diameter was measured in the most dilated MPDs. The MN heights were evaluated using EUS and were defined as the vertical distance from the top of MNs to the septum from which MNs arose [13].

Definitions of focal pancreatic parenchymal atrophy (FPPA) and upstream pancreatic atrophy (UPA)

Using preoperative CECT images, we firstly identified partial pancreatic parenchymal loss and fatty replacement as a FPPA candidate. The maximum and minimum diameters of the candidate were measured, and this was defined as FPPA when the product of these diameters exceeded 50 mm according to the report of Nakahodo et al [9]. FPPA was classified into the three morphological patterns: (1) cave-in type (unilateral atrophy), (2) slimness type (long-segment and irregular atrophy), and (3) slit type (wedge-shaped atrophy) [10].

On the other hand, upstream parenchymal atrophy (UPA) was defined as having a width of 4 mm or less from the MPD wall in the previous report [11]. Two gastroenterologists (S.H. and K.S.) reviewed the above-mentioned evaluations related to FPPA/UPA using preoperative CT images. Any discrepancies between our assessments were resolved by consensus after discussion. Interobserver agreement for image findings was calculated using the weighted kappa statistic.

Histological evaluations

According to the WHO classification [14], we histologically classified IPMN lesions of the resected specimens into the following: IPMN with an associated invasive carcinoma (IC), high-grade IPMN (HG-IPMN), and low-grade IPMN (LG-IPMN). The former two types were malignant, and the latter one was benign. On the basis of the immunostaining findings, histological subtypes were classified into four types, namely, gastric, intestinal, pancreatobiliary (PB), and oncocytic type (including intraductal oncocytic papillary neoplasm) [14].

Statistical analyses

All statistical analyses were performed using EZR (Saitama Medical Center, Jichi Medical University, Saitama, Japan), which is a graphical user interface for R (The R Foundation for Statistical Computing, Vienna, Austria). More precisely, it is a modified version of R Commander designed to add statistical functions frequently used in biostatistics [15]. The Pearson chi-square test or the Fisher exact test was used for the categorical variables, whereas the Student's t-test or the Mann-Whitney U test was used for continuous variables. A p-value of <0.05 was considered statistically significant. Continuous variables that showed significant differences were converted into categorical variables by calculating the cut-off value using a receiver operating characteristic (ROC) curve.

## Results

Characteristics of the 19 patients

Detailed characteristics of the 19 patients are shown in Table 1. The mean age of patients was 70 ± 7 years, and eight of the 19 were male. Laboratory data revealed elevated serum CA19-9 levels only in one patient. From imaging studies, the median diameter of the MPD was 8 mm, ranging from 3 to 24 mm. Although only one patient with a slightly dilated MPD of 3 mm did not meet the criteria of MD-IPMNs according to the international consensus guidelines, we included this patient because a papillary tumor with mucin secretion was observed mostly inside the MPD using imaging studies, despite minimal MPD dilatation. Using the resected specimens of the 19 patients, histological grades were found to be IC for two (11%), HG-IPMN for ten (53%), and LG-IPMN for seven (37%). Histological subtypes were gastric for eight (44%), intestinal for six (33%), PB for three (17%), and oncocytic for one patient (6%).

**Table 1:** Baseline characteristics. AMY: serum amylase levels; LIP: serum lipase levels; CA19-9: serum carbohydrate antigen 19-9 levels; CEA: serum carcinoembryonic antigen levels; Ph: pancreatic head; Pb: pancreatic body; Pt: pancreatic tail; MPD: main pancreatic duct; FPPA: focal pancreatic parenchymal atrophy; UPA: upper pancreatic atrophy; MN: mural nodule; PD: pancreaticoduodenectomy; DP: distal pancreatectomy; TP: total pancreatectomy; IPMN: intraductal papillary mucinous neoplasm of the pancreas; IC: IPMN with an associated invasive carcinoma; HG-IPMN: high-grade IPMN; LG-IPMN: low-grade IPMN.

Baseline characteristics	n = 19
Age, mean ± SD, year	70 ± 7
Sex (male:female), n	8:11
Symptoms, n (%)	3 (15)
Diabetes mellitus, n (%)	6 (32)
History of acute pancreatitis, n (%)	3 (15)
Laboratory data	
AMY, median (range), IU/L (n = 17)	70 (40–140)
LIP, median (range), IU/L (n = 9)	51 (23–197)
CA19-9, median (range), U/mL (n = 17)	4.7 (2.1–133)
CEA, median (range), ng/mL (n = 17)	2.3 (1.2–41)
Image findings	
Location (Ph:Pb:Pt), n	9:6:4
Diameter of MPD, median (range), mm	8 (3–24)
FPPA, n (%)	4 (21)
UPA, n (%)	12 (63)
Height of MN, median (range), mm	6 (2–15)
Surgical procedure	
PD:DP:TP, n	8:8:3
Pathological findings	
Grade	
IC, n (%)	2 (11)
HG-IPMN, n (%)	10 (53)
LG-IPMN, n (%)	7 (37)
Histological subtype (n = 18)	
Gastric type, n (%)	8 (44)
Intestinal type, n (%)	6 (33)
Pancreatobiliary type, n (%)	3 (17)
Oncocytic type, n (%)	1 (6)

Characteristics of patients with focal pancreatic parenchymal atrophy (FPPA)

Among the 19 patients with MD-IPMNs, 12 (63%) showed UPA, whereas four (21%) showed FPPA. There were no patients in whom UPA and FPPA coexisted in the pancreas. Details of the four patients with FPPA are described in Table 2. Regarding interobserver agreement for the two types of atrophy using imaging findings, the kappa values were 0.683 for FPPA and 0.890 for UPA.

**Table 2:** Four cases of MD-IPMNs with FPPA. MD-IPMNs: main duct intraductal papillary mucinous neoplasms of the pancreas; FPPA: focal pancreatic parenchymal atrophy; Ph: pancreatic head; Pb: pancreatic body; LG-IPMN: low-grade IPMN; IC: IPMN with an associated invasive carcinoma; HG-IPMN: high-grade IPMN.

Patient no.	Location	FPPA pattern	Histological grade	Histological subtype
1	Ph	Slimness type	LG-IPMN	Gastric
2	Ph	Slimness type	LG-IPMN	Gastric
3	Ph	Slit type	IC	Pancreatobiliary
4	Pb	Cave-in type	HG-IPMN	Intestinal

To clarify the clinical characteristics of FPPA, the clinical and pathological factors were compared between FPPA and UPA patients. Using univariate analysis, only the diameter of the MPD significantly differed between the two groups (median MPD diameter: 6 mm vs. 11 mm; p = 0.017 (Mann-Whitney U test); the difference of the medians, 5.5 mm (95% CI, 3.2-7.8 mm, Hodges-Lehmann estimate), Table 3). In addition, we analyzed an appropriate cut-off value of the MPD diameter to predict the presence of FPPA by using an ROC curve, and an MPD diameter of <7 mm was determined to be an appropriate cut-off value to diagnose FPPA.

**Table 3:** Univariate analysis of the characteristics of the MD-IPMN cases with FPPA and those with UPA. MD-IPMNs: main duct intraductal papillary mucinous neoplasms of the pancreas; FPPA: focal pancreatic parenchymal atrophy; UPA: upper pancreatic atrophy; AMY: serum amylase levels; LIP: serum lipase levels; CA19-9: serum carbohydrate antigen 19-9 levels; CEA: serum carcinoembryonic antigen levels; Ph: pancreatic head; Pbt: pancreatic body and tail; MPD: main pancreatic duct; MN: mural nodule. *Student's t-test; **Fisher's exact test; ***Mann-Whitney U test. P <0.05 was considered statistically significant.

	FPPA (n = 4)	UPA (n = 12)	P-value
Age, mean ± SD, year*	72 ± 11	70 ± 6	0.752
Sex (male:female), n**	1:3	7:5	0.569
Symptoms, n (%)**	1 (25)	2 (17)	1.000
Diabetes mellitus, n (%)**	3 (75)	3 (25)	0.118
History of acute pancreatitis, n (%)**	1 (25)	2 (17)	1.000
Laboratory data			
AMY, median (range), IU/L, (n = 4/11)***	61 (46–140)	60 (44–144)	1.000
LIP, median (range), IU/L, (n = 1/6)***	150	45 (23–129)	0.286
CA19-9, median (range), U/mL, (n = 4/11)***	15.4 (2.1–133)	3.8 (2.1–11)	0.114
CEA, median (range), ng/mL, (n = 4/11)***	2.7 (1.4–25)	2.3 (1.2–41)	0.647
Image findings			
Location (Ph:Pbt), n**	3:1	4:8	0.262
Diameter of MPD, median (range), mm***	6 (3–8)	11 (7–24)	0.017
Diameter of MPD < 7 mm, n (%)**	3 (75)	0	0.007
Height of MN, median (range), mm***	3 (0–6)	7 (2–15)	0.074
Pathological findings			
Diagnosis (benign:malignant), n**	2:2	4:8	0.604
Histological subtype (gastric:non-gastric), (n = 4/11)**	2:2	5:6	1.000

The relationship between the lateral spreading of MD-IPMN lesions and FPPA

**Figure 2:**
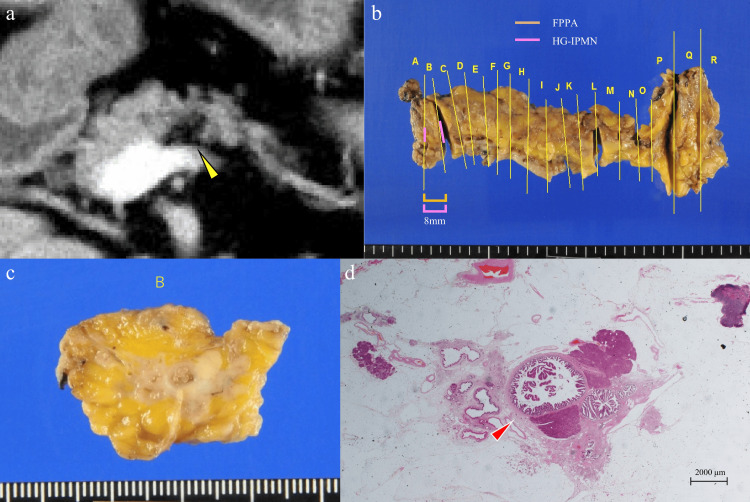
Case involving the extension of the MD-IPMN lesion concordant with that of FPPA. An 80-year-old woman was diagnosed with cave-in type FPPA in the pancreatic body (a, arrowhead). Preoperative endoscopic ultrasonography showed a suspected intraductal small lesion in the MPD of the pancreatic body coexisting with FPPA, indicating a suspected small MD-IPMN. Pancreatic juice cytology provided the diagnosis of adenocarcinoma (MUC2 positive). From the histology of the resected specimens, a papillary atypical epithelium was detected mainly in the MPD (d, arrowhead) and partially in the branch ducts of the pancreatic body. The pancreatic parenchyma at the site of the MD-IPMN lesion was markedly atrophic and replaced by adipose tissue (b)–(d). Due to the detection of diffusely MUC2-positive atypical epithelium and the lack of the invasive component, the histological diagnosis was intestinal-type HG-IPMN. (a) Contrast-enhanced computed tomography (horizontal image). (b) Mapping of tumor extension. (c) Resected specimens of the primary lesion. (d) Histological evaluations of resected specimens. Hematoxylin and eosin staining (original magnification ×1.25). FPPA: focal pancreatic parenchymal atrophy; HG-IPMN: high-grade IPMN; MD-IPMN: main duct intraductal papillary mucinous neoplasm of the pancreas; MPD: main pancreatic duct; MUC2: mucin 2 glycoprotein.

For four MD-IPMN patients involving FPPA in their pancreas, we examined whether or not the lateral spreading of the MD-IPMN lesions was histologically concordant with that of FPPA. Only in one patient was the extension of the MD-IPMN lesion concordant with the location of the FPPA (Figure 2).

**Figure 3:**
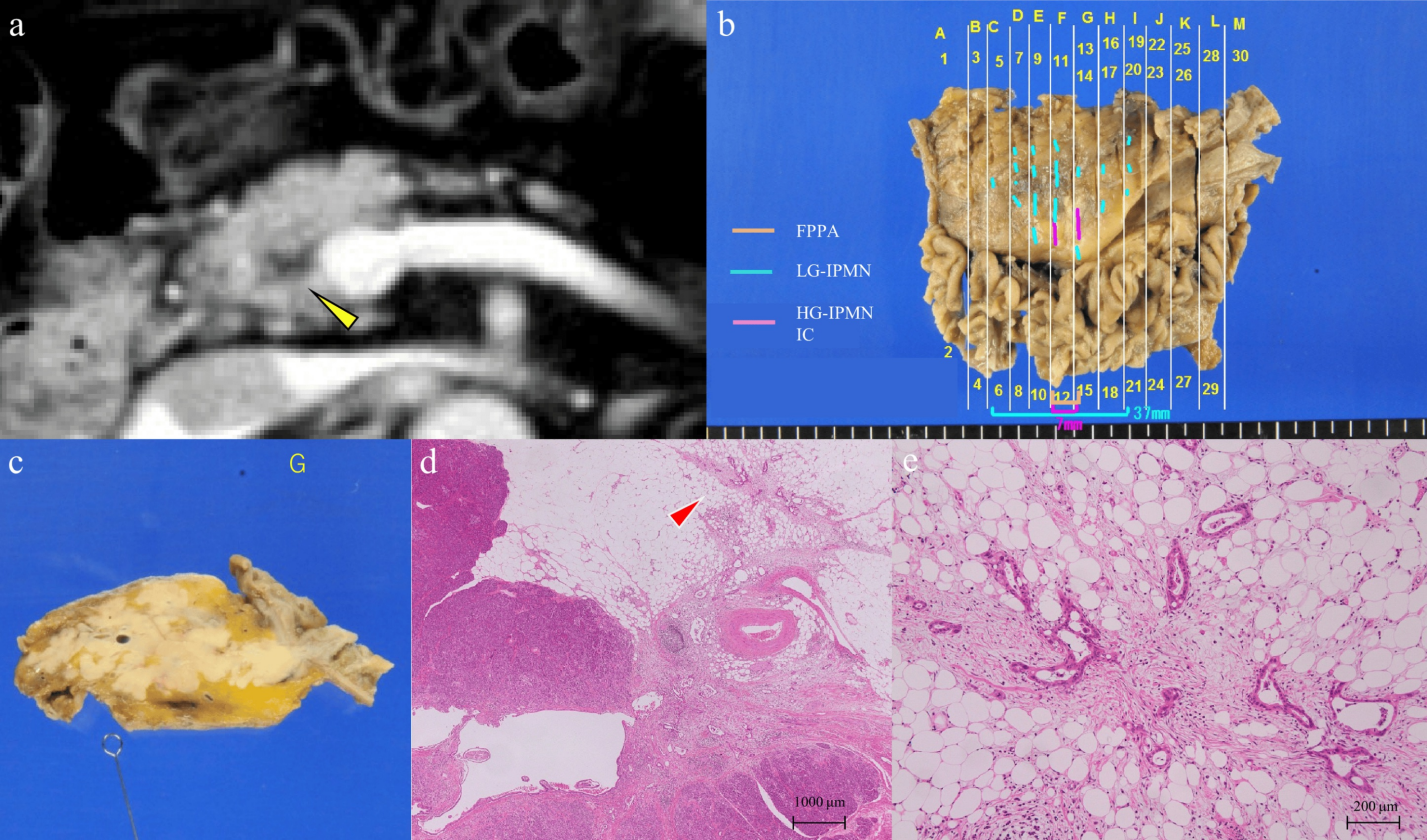
Case with the extension of the MD-IPMN lesion beyond that of FPPA. A 74-year-old man was diagnosed with slit-type FPPA in the pancreatic head (a, arrowhead). The lateral spreading of MD-IPMN, which measured 37 mm along the main pancreatic duct (b), extended beyond the location of FPPA. However, most of the lesions that spread were LG-IPMN, and the component of HG-IPMN and IC was located only in the center of the MD-IPMN lesion (b). Atrophic pancreatic parenchyma with fat replacement was histologically observed only in the part of the pancreas involving this malignant component (c)–(e). (a) Contrast-enhanced computed tomography (horizontal image). (b) Mapping of tumor extension. (c) Resected specimens of the primary lesion. (d) and (e) Histological evaluations of resected specimens. Hematoxylin and eosin staining ((d), original magnification ×2) and ((e), original magnification ×10). FPPA: focal pancreatic parenchymal atrophy; HG-IPMN: high-grade IPMN; IC, IPMN with an associated invasive carcinoma; LG-IPMN: low-grade IPMN; MD-IPMN: main duct intraductal papillary mucinous neoplasm of the pancreas.

In another patient, the lateral spreading of the MD-IPMN lesion extended beyond the location of the FPPA (Figure 3). However, the FPPA was histologically observed only in the part of the pancreas involving a malignant component.

**Figure 4:**
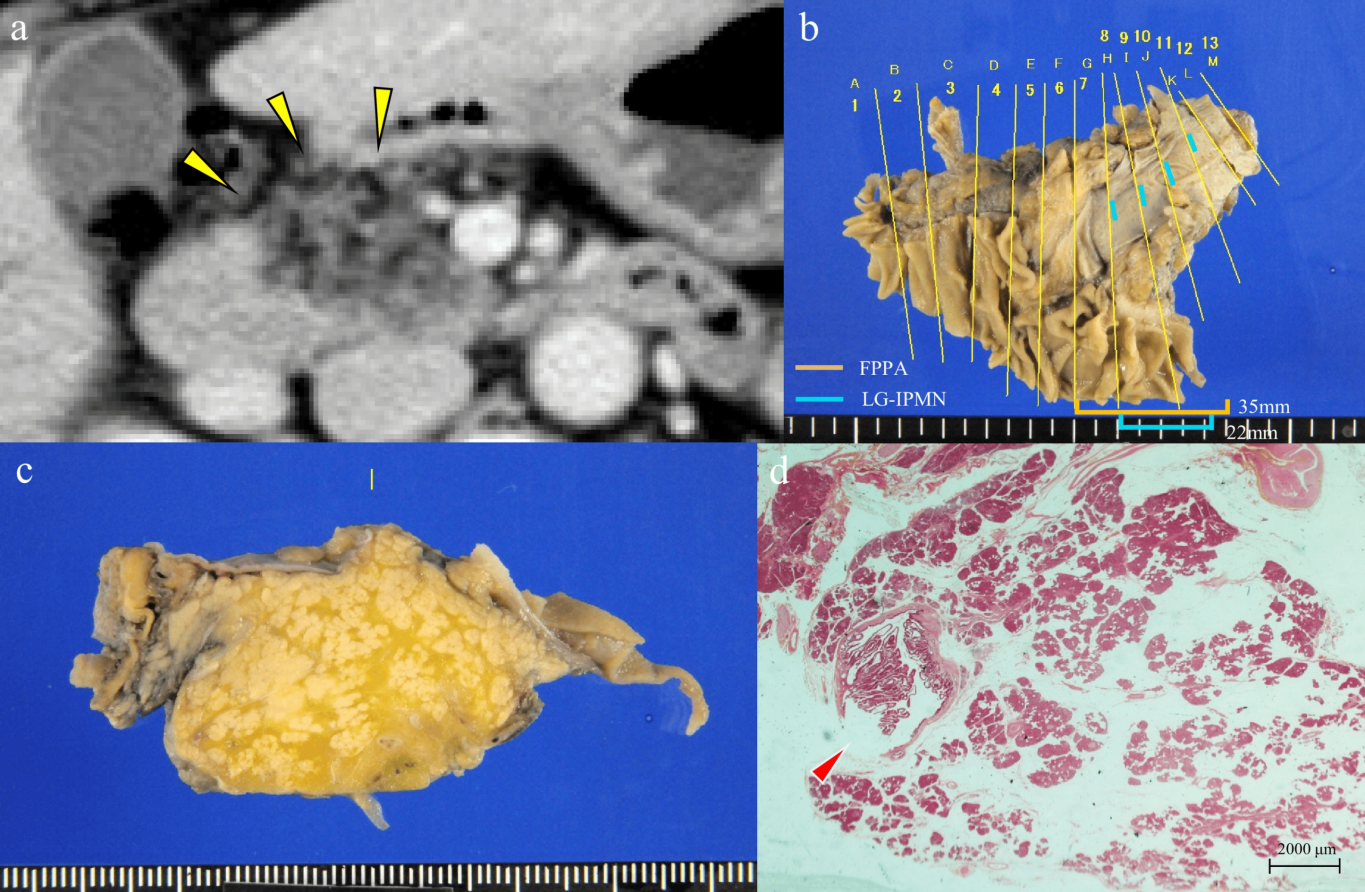
Case with the extension of the MD-IPMN lesion within the area of the FPPA. A 55-year-old woman was diagnosed with slimness-type FPPA in the pancreatic head (a, arrowhead). The histological diagnosis was determined as gastric-type LG-IPMN, which measured 22 mm along the main pancreatic duct. Pancreatic atrophy was observed in an extensive area in the resected specimens, and MD-IPMN was located within the area of the FPPA. (a) Contrast-enhanced computed tomography (horizontal image). (b) Mapping of tumor extension. (c) Resected specimens of the primary lesion. (d) Histological evaluations of resected specimens. Hematoxylin and eosin staining (original magnification ×1.25). FPPA: focal pancreatic parenchymal atrophy; LG-IPMN: low-grade IPMN; MD-IPMN: main duct intraductal papillary mucinous neoplasm of the pancreas.

For the other two patients, their MD-IPMN lesions were located within the MPD of the pancreatic part involving FPPA (Figure 4).

## Discussion

Among the 19 patients with surgically resected MD-IPMNs without apparent invasive components, FPPA was detected in four patients (21%), two of whom (50%) had malignant MD-IPMNs. Although the location of the FPPA and MD-IPMN in the pancreas exactly matched only in 25% of the patients (1/4), most of the MD-IPMN lesions histologically occurred within the pancreatic part involving FPPA. Therefore, despite the low detection rate of FPPA among MD-IPMN patients, FPPA roughly indicates the position of the MD-IPMN lesion in the pancreas. In addition, since an MPD diameter of <7 mm was shown to be an indicator of FPPA from a comparison with patients having UPA, we speculate that FPPA may be a sign of the initial phase of MD-IPMN before the MPD enlarges and UPA appears.

Recently, FPPA has been recognized as an indicator of pre-cancerous lesions (low-grade PanINs) [16] or cancers at an early stage (high-grade PanINs or small PDACs) [9-12,17], which may mean that FPPA is a sign of the existence of intraductal epithelial neoplasms in the pancreas. FPPA is thought to develop due to several factors, such as asymptomatic focal inflammation due to the stenosis/obstruction of the MPD or branch pancreatic ducts and the microenvironment changes caused by the cancer lesions [10,11]. On the other hand, UPA is usually observed in advanced-stage PDACs, and the mechanism may be related to the massive stenosis/obstruction of the MPD involving upstream MPD dilation. Considering these, FPPA and UPA may be continuous imaging findings according to the progression from pre-cancerous, early-stage to advanced-stage lesions among patients with PDACs [10,17].

On the other hand, although IPMNs, including MD-IPMNs, are intraductal epithelial neoplasms as well as PanINs, there is only a case report on FPPA in MD-IPMNs, which could be obtained from a search of English and Japanese literature from 2006 to 2023 using ‘MEDLINE’ and ‘Ichushi web’ databases [18]. Under the circumstances, we aimed to examine the detection rate and clinical characteristics of FPPA in MD-IPMNs. From the results of the present study, including MD-IPMN patients, FPPA was observed in only 21% of patients (4/19), whereas UPA was detected in >50% of the population, meaning that UPA may be a more general pattern of pancreatic parenchymal atrophy than FPPA among MD-IPMN patients. In addition, only the MPD diameter significantly differed between MD-IPMN patients with FPPA and those with UPA. Since the MPD diameter is associated with the degrees of advancement and/or progressive nature of MD-IPMN lesions [3], we hypothesized that the pattern of pancreatic parenchymal atrophy in MD-IPMNs may be sequential, as well as that in PDAC/PanIN. Although time series CT images of the respective MD-IPMN patients are needed to verify this hypothesis, those CT images were not available for all patients included in this study. If this hypothesis is correct, the difference in the detection rate for MD-IPMN patients with FPPA and those with UPA may be influenced by a selection bias caused by the use of patients who underwent surgery. In other words, detecting MD-IPMNs involving FPPA may be difficult due to inconspicuous imaging findings compared to MD-IPMNs involving UPA. On the other hand, the MPD diameter in patients with MD-IPMN appears to be influenced by other factors, such as the degree of mucin secretion and the degree of advancement and/or progressive nature. Therefore, the relationship between MD-IPMNs with FPPA and those with UPA may not necessarily be sequential in the pathogenesis of MD-IPMNs, and the pathogenic pathways for these two types of MD-IPMNs may differ, unlike the pathogenesis of the pancreatic parenchymal atrophy in the population with PDACs.

For the determination of the surgical procedure for MD-IPMNs, it is important to evaluate the lateral spreading of the IPMN lesions to the estimated resection margin along the MPD. Although peroral pancreatoscopy can be used to determine the resection margin of MD-IPMN lesions, the diagnostic value was not similar among the reports [4,19,20]. In addition, there are still issues related to peroral pancreatography, such as high cost and post-endoscopic retrograde cholangiopancreatography (ERCP) pancreatitis. On the other hand, since FPPA has been reported as a useful image finding to predict the extension of the lateral spreading of PanIN [10], we hypothesized that FPPA might serve as a predictor of the extension of MD-IPMN lesions as well. Although the rate of complete consistency between the histological extension of FPPA and that of MD-IPMN lesions was 25% (n = 1) among four MD-IPMN patients involving FPPA, most of the MD-IPMN lesions of those patients were located nearly within the MPD of the pancreatic part involving FPPA. Thus, FPPA may roughly indicate the location of MD-IPMN lesions, although it is not as good for FPPAs involving MD-IPMN lesions as that involving PanIN lesions. It may be due to the effect of mucin production on the pancreatic parenchyma, since the positional consistency between FPPA and MD-IPMN lesions was somewhat inaccurate compared to FPPA involving PanIN lesions. In any case, since FPPA was the first sign of a small MD-IPMN lesion (<5 mm) in one patient (patient no. 4 in Table 2), this finding might contribute to the detection of small/possibly early-phase MD-IPMNs as well as PanINs/early-stage PDACs. However, since this pattern of pancreatic parenchymal atrophy appears a slightly inaccurate predictor of the extension of MD-IPMN lesions, further studies are needed.

This study has some limitations. First, this was a single-center retrospective study for patients who had undergone surgical treatment, possibly causing a selection bias. Second, the sample size in this study was small, which made multivariate analysis unfeasible. Therefore, more patients may be needed to verify the results of this study. Third, the procedures for CT imaging, such as slice thickness, varied over time, and longitudinal imaging data were not available. Fourth, the definitions of pancreatic parenchymal atrophy are complex and different across previous reports. Although these limitations exist, this study provides the clinical possibility for acquiring imaging findings for MD-IPMN patients.

## Conclusions

FPPA was detected in approximately 20% of resected MD-IPMNs without apparent invasive carcinoma and may be an indicator of MD-IPMN lesions biologically related to mild MPD dilation (<7 mm), such as an early stage of the disease and less mucin secretion. In addition, although the clinical usage may be limited, FPPA might provide a clue for the localization of small MD-IPMNs.
